# Direct PCR with the CDC 2019 SARS-CoV-2 assay: optimization for limited-resource settings

**DOI:** 10.1038/s41598-022-15356-7

**Published:** 2022-07-11

**Authors:** Christia M. Victoriano, Megan E. Pask, Nicole A. Malofsky, Adam Seegmiller, Steve Simmons, Jonathan E. Schmitz, Frederick R. Haselton, Nicholas M. Adams

**Affiliations:** 1 Department of Biomedical Engineering, Vanderbilt University, 5923 Stevenson Center, 1225 Stevenson Center Lane, Nashville, TN 37240 USA; 2 Department of Pathology, Microbiology and Immunology, Vanderbilt University Medical Center, Nashville, TN USA; 3 Bioventures, Inc., Murfreesboro, TN USA; 4 Molecular Infectious Diseases Laboratory, Vanderbilt University Medical Center, Nashville, TN USA; 5 Vanderbilt Institute for Infection, Immunology, and Inflammation, Vanderbilt University Medical Center, Nashville, TN USA; 6 Department of Chemistry, Vanderbilt University, Nashville, TN USA; 7 Thermo Fisher Scientific, 180 Oyster Point Blvd, South San Francisco, CA 94080 USA; 8 Department of Biomedical Engineering, Columbia University, 351 Engineering Terrace, 1210 Amsterdam Avenue, New York, NY 10027 USA

**Keywords:** Microbiology techniques, Assay systems

## Abstract

PCR-based diagnostics generally require nucleic acid extraction from patient specimens prior to amplification. As highlighted early in the COVID-19 pandemic, extraction steps may be difficult to scale during times of massive demand and limited reagent supply. Forgoing an extraction step, we previously reported that the N1 primer/probe-set of the widespread CDC COVID-19 assay maintains high categorical sensitivity (95%) and specificity (100%) with direct inoculation of viral transport media (VTM) into qRT-PCR reactions. In contrast, the N2 set demonstrated a prominent C_t_ delay and low sensitivity (33%) without extraction. In the current study, we have improved the performance of this modified CDC assay (in particular the N2 set) by incorporating N1/N2/RNase P multiplexing and dissecting the effects of annealing temperature, VTM interference, and inoculum volume. The latter two factors exerted a more prominent effect on the performance of N2 than N1, although these effects were largely overcome through elevated annealing temperature. This unextracted/multiplex protocol was evaluated with 41 SARS-CoV-2 positive and 43 negative clinical samples, demonstrating a categorical sensitivity of 92.7% and specificity of 100% *versus* the unmodified CDC methodology. Overall, this work offers a generalizable strategy to maximize testing capabilities for COVID-19 or other emerging pathogens when resources are constrained.

## Introduction

The coronavirus disease 2019 (COVID-19) pandemic has highlighted the need for rapid and resource-efficient molecular diagnostics that facilitate effective patient management and contain the spread of the causative SARS-CoV-2 virus and its emergent variants^1^. At the onset of the pandemic, several qRT-PCR assays for SARS-CoV-2 detection were rapidly developed by the United States Centers for Disease Control and Prevention (CDC), as well as other government organizations, commercial entities, and diagnostic laboratories worldwide^2, 3^. Because of the respiratory nature of SARS-CoV-2, testing from the onset focused primarily on nasopharyngeal specimens^4^, the specimen-of-choice for other respiratory infections such as influenza, parainfluenza, rhinovirus, and other coronaviruses^5–8^. In this standardized workflow, prior to qRT-PCR, RNA is first extracted from the samples using a variety of common commercial kits^4^. Most of these original assays required singleplex detection of multiple viral targets within separate wells of an qRT-PCR plate, along with an internal control to ensure adequate specimen collection, extraction, and thermocycling. Specifically, the original CDC-developed assay entailed separate reactions targeting three loci of the virus’s nucleocapsid (N) gene (N1, N2, and N3, the latter was subsequently eliminated) and a fourth control reaction targeting human RNase P (RP)^2^. Not uncommonly, initial strategies for assay development incorporated conservative designs—such as multiple singleplex amplifications—to hasten test deployment and improve specificity, even if they decreased overall throughput^9^.

A practical advantage of the CDC assay (and similar assays) is that its oligonucleotide sets can be flexibly incorporated with a variety of extraction and amplification reagents/instruments to yield a composite methodology. This is not the case for most commercially developed COVID-19 assays, whose pre-packaged nature can lead to higher per-test costs and a reliance on the manufacturer’s supply chain to ensure sufficient testing throughput. At the same time, the CDC-style assays require integration of individual manual steps, with more significant labor-needs and reduced assay throughput. Additionally, as the initial months of the pandemic demonstrated, the supply of basic molecular extraction reagents themselves became assay limiting when faced with massive global demand. In many respects, an ideal COVID-19 testing method, especially for resource-limited settings, would combine the flexibility and cost of a non-commercial qRT-PCR assay with an increase in speed and scalability.

To this end, we have previously reported that the CDC assay could be deployed without a dedicated RNA extraction procedure^10^. In this work, we compared standard clinical-use CDC procedures to the direct pipetting of heat-inactivated viral transport medium (VTM) sample into the qRT-PCR reaction. Across 81 clinical samples, categorical positive agreement was high for N1 (95%), N3 (95%), and RP (100%) primer/probe sets. Nonetheless, amplification for the unextracted N2 primer set amplification was significantly inhibited, as evidenced by significantly higher C_t_ values and a positive agreement of only 33%. Even without a functional N2 primer set, this direct approach appeared viable due to the analytic performance of the other nucleocapsid targets. But the failure of the N2 primer/probe set remains puzzling, as the assay risks a theoretical breakdown in specificity without a functional N2 reaction. Moreover, the presence of multiple independent targets helps ensure assay integrity against emergent variants with sequence polymorphisms in one of these regions^11–15^.

In this study, we sought to explain the apparent discrepancy among CDC primer sets for direct pipetting of unextracted heat-inactivated viral transport medium (VTM) samples into qRT-PCR reactions. We developed general procedures for optimizing the qRT-PCR performance following direct inoculation with raw sample matrix, including annealing temperature optimization, volume optimization, and interference analysis. We apply these optimization methods to maximize the performance of the CDC primer sets using unextracted specimens, combine the optimized reactions into a multiplexed qRT-PCR assay for SARS-CoV-2, and compare the performance of this design to the standard CDC methodology using samples from COVID-19 patients.

## Results

### Effect of VTM components on N1 and N2 on overall performance

**Figure 1:**
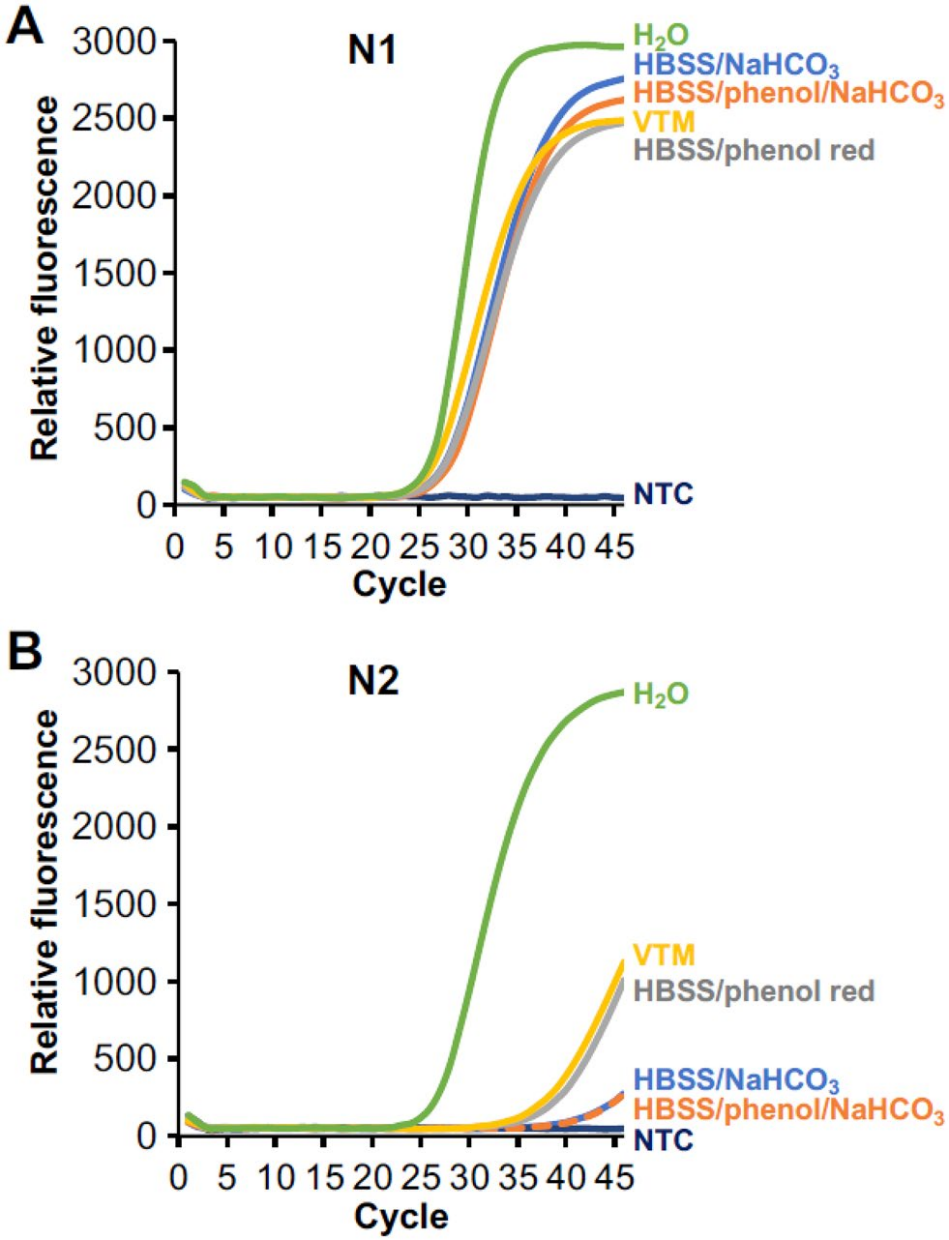
Comparison of the qRT-PCR performance of the (**A**) N1 and (**B**) N2 primer set with VTM and similar media added using the CDC SARS-CoV-2 qRT-PCR protocol. N2 reactions are negatively impacted for all media tested. Each curve is the average of two experiments (N = 2).

Every VTM mixture tested had very little impact on the PCR performance of the N1 primer set but a major effect on that of N2. As Fig. 1 illustrates the qRT-PCR curves corresponding to water alone are similar for both N1 and N2 (green lines) and both have a C_t_ of around 25. In the case of N1, VTM and all the other comparison VTM cocktails (HBSS/phenol red (grey line), HBSS/NaHCO_3_ (blue), HBSS/phenol red/NaHCO_3_ (orange)) also have a C_t_ close to 25 (Fig. 1A). However, for N2, considerable delay in C_t_ is observed for all media (Fig. 1B).

### Effect of VTM on optimal primer annealing temperature

**Figure 2:**
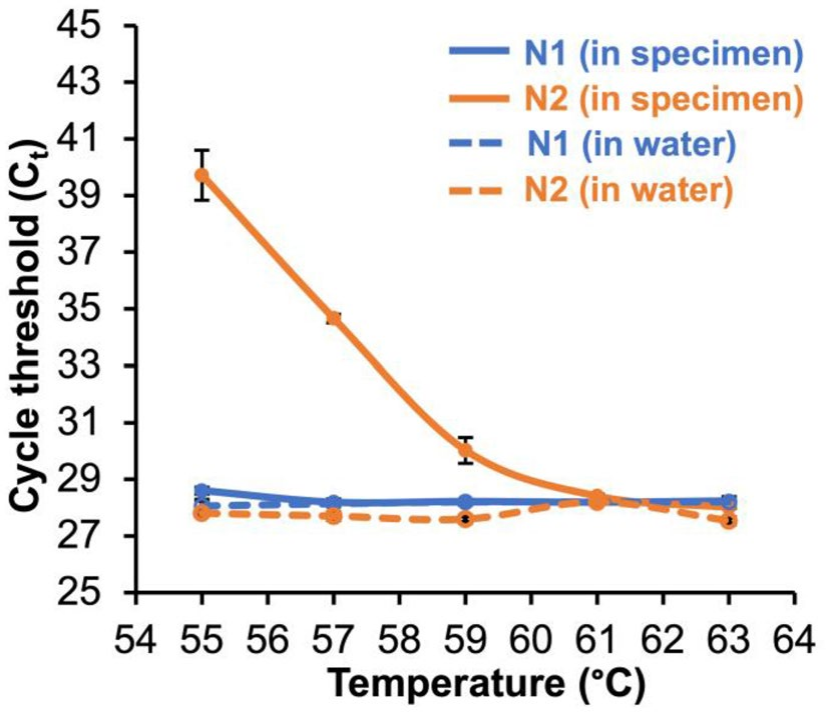
Annealing temperature has a small effect on the C_t_s of N1 and N2 using a pooled SARS-CoV-2 positive specimen spiked into water (blue and orange dotted lines, respectively) or for N1 when spiked into a pooled negative specimen (blue solid line), but annealing temperature has a large effect on the C_t_s of N2 when a pooled positive specimen is spiked into a pooled negative specimen (orange solid lines). Mean ± s.d. (N = 4).

A differential effect for N1 and N2 was observed for changes in annealing temperature for VTM but not water. For five common primer sets reported in the literature (i.e., N1, N2, N3, E, and RdRp) using SARS-CoV-2 RNA spiked into VTM (Supplemental Fig. 1) and with the primer sets used in the CDC SARS-CoV-2 protocol (i.e., N1, N2) using pooled positive clinical specimens spiked into pooled negative clinical specimens (Fig. 2), there was greater C_t_ variation across the 10 °C range of annealing temperatures compared to reactions with RNA or positive specimens spiked into water. It was observed that C_t_s for reactions in both VTM and pooled negative clinical specimens at lower annealing temperatures showed the greatest C_t_ delays when compared to the corresponding reactions in water. At the annealing temperature of 55 °C specified by the CDC, C_t_s for N1 and N2 were delayed on average by 3.1 cycles for N1 and 13.2 cycles for N2, respectively (Supplemental Fig. 1), due to the presence of VTM. At an annealing temperature of 61 °C, C_t_s for both targets were similar in VTM and water, with cycle delays of − 0.1 for N1 and − 0.5 for N2 (Supplemental Fig. 1). In pooled positive clinical samples spiked into either pooled negative specimens or water at an annealing temperature of 55 °C, the average C_t_ delay due to the presence of patient specimen was 0.5 for N1 and 11.9 for N2, whereas at an annealing temperature of 61 °C, the average C_t_ delay due to the presence of patient specimen was only − 0.2 for N1 and 0.2 for N2 (Fig. 2). The optimal annealing temperature found for N1 and N2 in VTM was 61 °C. The C_t_s for RNase P were similar between 59 – 64 °C (data not shown), thus 61 °C was chosen as the optimal annealing temperature for the optimized unextracted assay.

### Effect of annealing temperature on sodium interference

**Figure 3:**
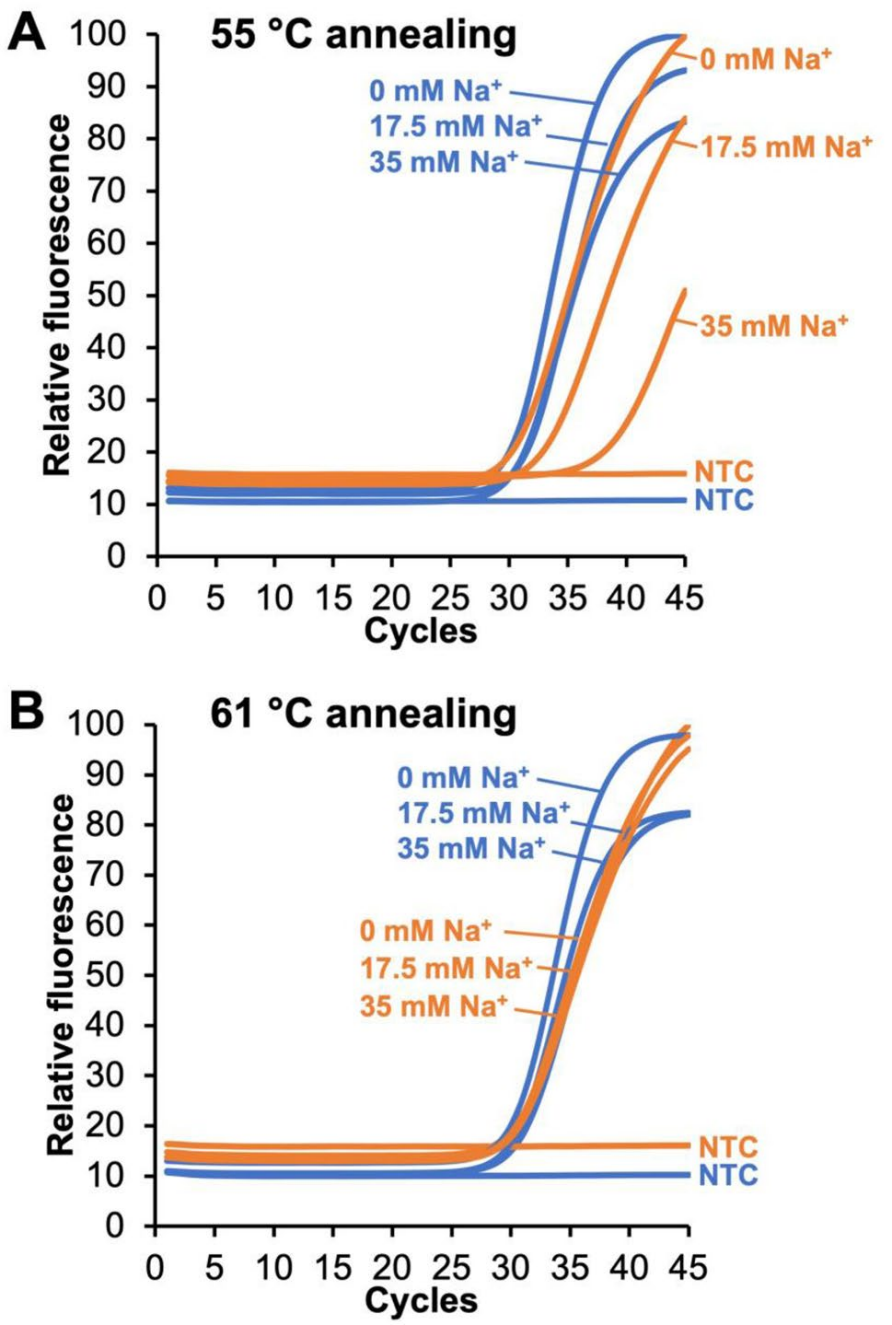
Effect of sodium concentration and annealing temperature on the qRT-PCR performance of N1 and N2 reactions. (**A**) At an annealing temperature of 55 °C, increasing sodium has no effect on N1 performance (blue curves) but a major impact on N2 performance (orange curves). (**B**) At an annealing temperature of 61 °C, however, increasing sodium has no effect on N1 (blue curves) or N2 performance (orange curves). Each curve is the average of four experiments (N = 4).

The direct effect of sodium, a major component of VTM, on the reaction performance at both 55 °C and 61 °C was overcome by the optimized annealing temperature. As shown in Fig. 3A, similar to the VTM mixtures at 55 °C, final sodium concentration in the reaction also had a measurable impact on reaction performance for N2 but not N1. C_t_ increased with concentration of salt indicating that sodium has a major effect on the performance of the N2 primer set. In fact, considering the salt concentration of VTM itself (~ 140 mM Na^+^ in the HBSS base and ~ 35 mM Na^+^ in the reaction tube), sodium seems to be a major inhibitor of N2 performance and is present when VTM is added directly to the reaction mixture as part of the unextracted protocol. At 61 °C, however, the effect of sodium on N2 performance is unaffected (Fig. 3B), indicating that the negative effect of sodium, a major N2 inhibitor in VTM, is temperature dependent. Taken together, these results indicate that changing the CDC’s prescribed annealing temperature from 55 to 61 °C may resolve the issues with N2 in unextracted clinical specimens.

### Effect of VTM volume added to PCR reaction

**Figure 4:**
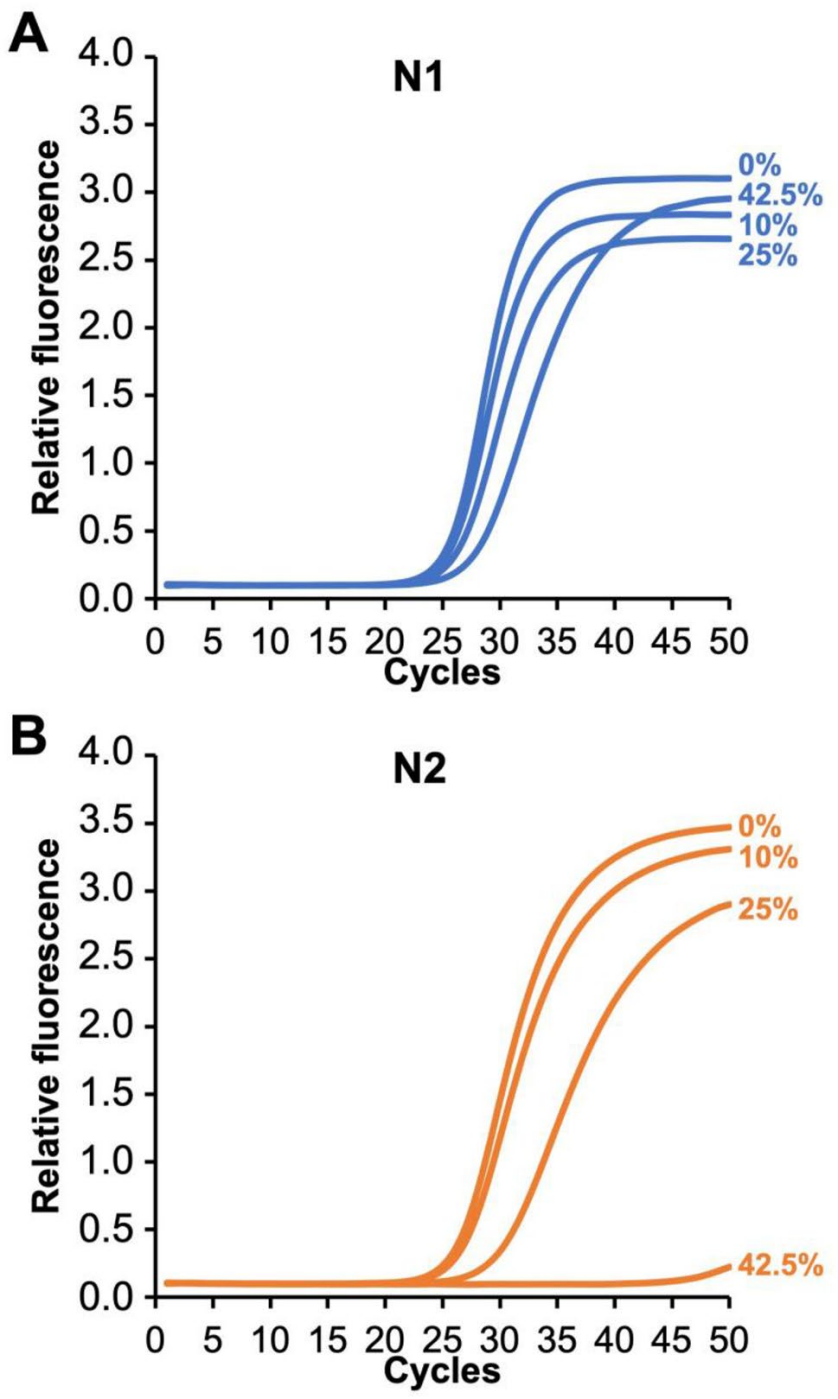
Effect of VTM volume added to an qRT-PCR reaction on performance of primer sets N1 (**A**) and N2 (**B**). The addition of 25% VTM was selected as a reasonable balance between the RNA copies introduced to the reaction and the inhibition from VTM for the two primer sets. Each curve is the average of four experiments (N = 4).

Both N1 and N2 were impacted by adding increasing amounts of VTM to the qRT-PCR reaction. As shown in Fig. 4, the addition of an increasing VTM volume to a fixed number of spiked targets (5 × 10^3^ copies/reaction) into VTM has an effect on both primer sets using an annealing temperature of 61 °C. The results for N1 show virtually no delay in C_t_ at 10% and 25% VTM and a slight delay in C_t_ with 42.5% VTM added to the reaction (Fig. 4A). On the other hand, the results for N2 show almost no effect on C_t_ at 10% VTM, a C_t_ delay of ~ 4 cycles at 25%, and complete qRT-PCR failure at 42.5%. The addition of 25% VTM was selected as a reasonable balance between the RNA copies introduced to the reaction (2.5-fold more than 10%) and the inhibition from VTM for the two primer sets.

### Singleplex vs. multiplex primer set performance

**Figure 5:**
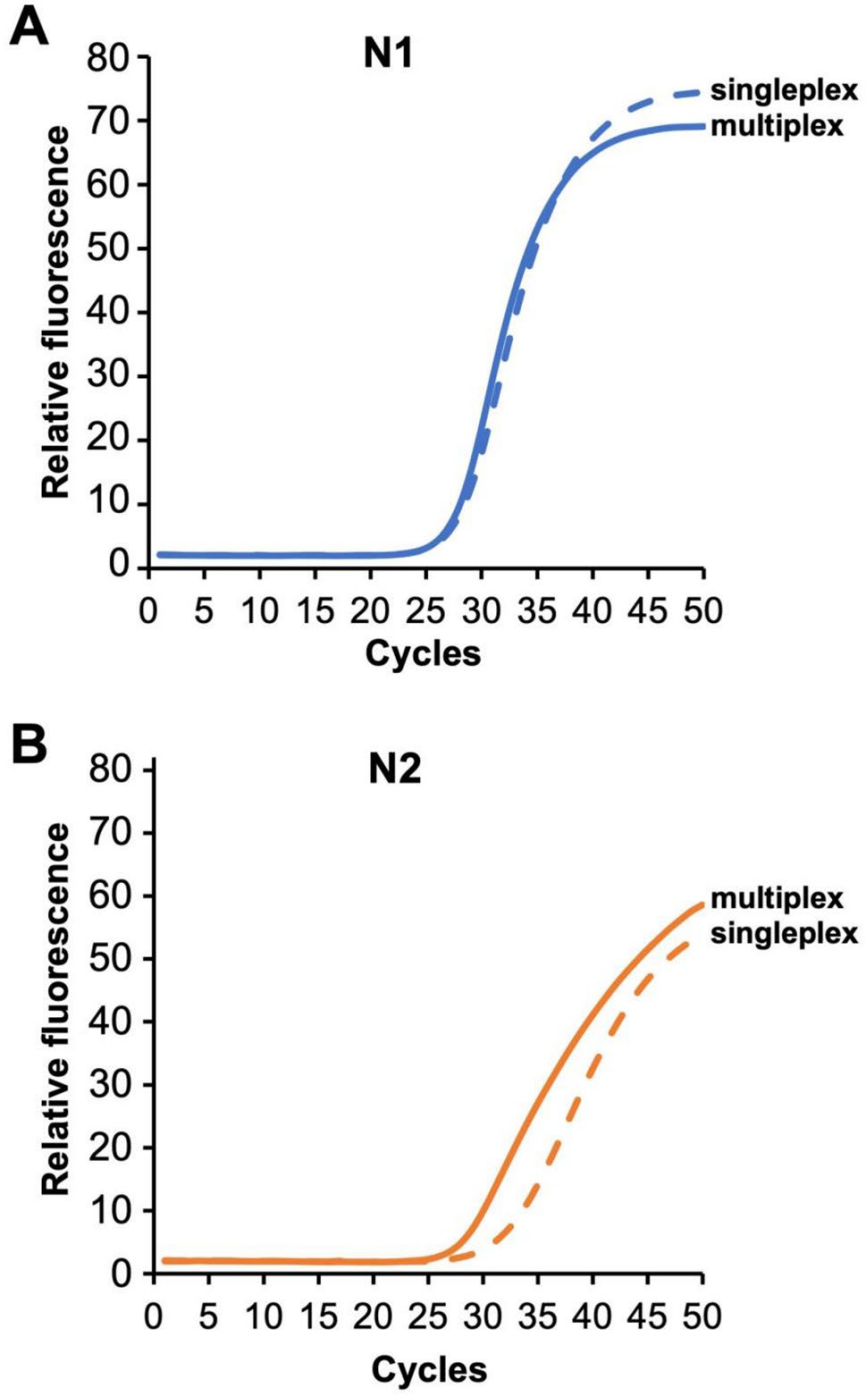
Comparison of singleplex and multiplex qRT-PCR with 5 µL of an unextracted positive patient sample added to the reaction. Each curve is the average of three experiments (N = 3).

Whether using surrogate samples of synthetic RNA spiked into VTM (see Supplemental Fig. 2) or using pooled negative clinical samples spiked with a pooled positive specimen (Fig. 5), the performance of the singleplex reactions was preserved when combined into a multiplex reaction. When combined in multiplex, neither of the two N-gene targets exhibited a change in C_t_ when compared to the singleplex assay. As shown in Supplemental Fig. 2, both the multiplexed and the singleplex reactions amplified RNA at very low concentrations of synthetic RNA target spiked into VTM at 61 °C.

### Multiplex assay performance using clinical nasopharyngeal samples in VTM

C_t_ values for N1, N2, and RNase P were delayed in our unextracted assay when compared to the clinical test results using the CDC assay (Δ*C*_t_ = 4.4 ± 0.5, 5.8 ± 0.4, and 6.1 ± 0.9, respectively; see Table 1), although delays for the N2 target were substantially less at the optimized annealing temperature in this assay when compared to our previous study^10^. When compared to the gold standard CDC assay results, overall sensitivity of 92.7% and specificity of 100% were observed for our assay with unextracted samples compared to clinical testing using extracted samples (Table 1). N1 and N2 targets both demonstrated 92.7% positive percent agreement (95% confidence interval, Wilson score method: 80.0–98.5%) and 100% negative percent agreement (95% confidence interval: 91.8–100%) with the predicate clinical result. RNase P demonstrated 86.9% positive percent agreement (95% confidence interval: 78.1–92.5%) with the clinical result (negative agreement not applicable for RP).

**Table 1:** Comparison of SARS-CoV-2 detection from unextracted clinical nasopharyngeal specimens using our multiplexed assay optimized for direct qRT-PCR of unextracted specimens compared to the original clinical diagnostic test results using the FDA emergency-use authorized CDC singleplex assay.

Primer/Probe	Positive percent agreement	ΔC_t_	Negative percent agreement
N1	92.7% (38/41)	4.4 ± 0.5	100% (43/43)
N2	92.7% (38/41)	5.8 ± 0.4	100% (43/43)
RP	86.9% (73/84)	6.1 ± 0.9	NA

**Figure 6:**
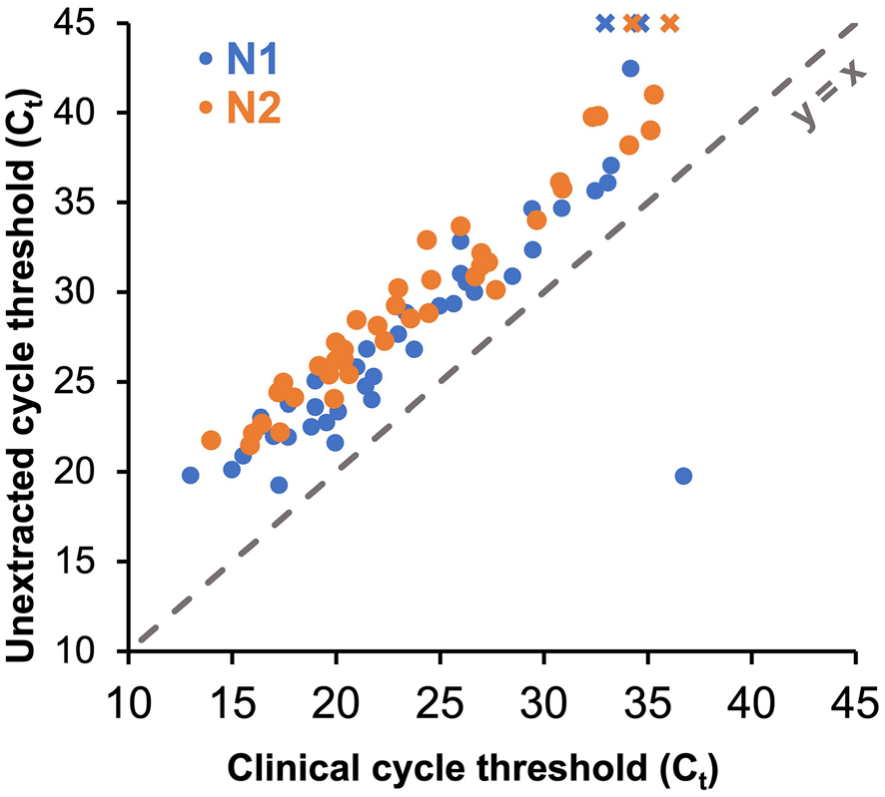
Comparison of C_t_ values from the original clinical test result to our optimized unextracted multiplex test. The dotted line represents the theoretical trendline of a perfect match. The data points represented by the “x” symbols were not detected (i.e., did not amplify within 45 cycles).

Figure 6 compares the clinical C_t_ values obtained with singleplex reactions for N1 and N2 and the corresponding C_t_ for N1 and N2 obtained in our optimized multiplex reaction. With the exception of one outlier, the data shows relatively constant ΔC_t_ across the range of C_t_ values.

## Discussion

We have demonstrated a resource-optimized clinical diagnostic workflow based on direct addition of 5 µL of a nasopharyngeal VTM sample to a qRT-PCR reaction that reduces time, cost, and labor of the original CDC 2019 SARS-CoV-2 Assay by (1) replacing the traditional multi-step RNA extraction process with a simple heat-inactivation sample preparation strategy and (2) combining the N1, N2 and RNase P primer/probe sets into a single multiplexed reaction (refer to the comparison schematic in Supplemental Fig. 3). This direct PCR approach achieved a categorical sensitivity of 92.7% and specificity of 100% for N1 and N2 according to the CDC’s criteria for identifying a positive result (Table 1). These results were obtained by employing the same heat-inactivation protocol we reported previously^10^, but by simply changing the reaction annealing temperature from 55 to 61 °C, we restored the previously impacted N2 primer set. These results were also obtained in a multiplexed design, demonstrating the potential for higher throughput and additional resource savings. Furthermore, this approach is easily scalable because large batches of tubes can be simultaneously heat-treated and more patient specimens can be analyzed on a single qPCR plate.

This workflow’s elimination of the RNA extraction step results in two major improvements over the original workflow. First, the need to perform the RNA extraction step in the CDC protocol is a significant bottleneck to meeting high throughput testing demands. Removing RNA extraction from the workflow eliminates the need for expensive instrumentation as well as specialized reagents which have posed critical issues in the current pandemic. Since RNA extraction accounts for nearly half of the total testing time, eliminating this step increases throughput. While the heat-inactivation step that replaces RNA extraction also requires some specimen handling and specimen-heating instrumentation, the protocol is much simpler and the resource burden is significantly lower. Chemical-based inactivation protocols have the potential to further reduce resource requirements, but chemical additives (e.g., detergents and enzymes) can negatively impact qPCR performance.

The second major improvement over the original workflow relates to the cost savings and throughput improvements of multiplexing. The current singleplex CDC workflow requires three qRT-PCR reactions instead of one, considerably increasing supply use, and in particular excess use of qRT-PCR enzyme kits which constitute the largest portion of reaction cost. In resource-limited settings, where access to these reagents and supplies are often restricted, a strategy in which the necessary reactions are multiplexed in a single well conserves resources, increases testing efficiency, and simplifies the operating procedure. This point is especially relevant in a global testing context. For instance, while reduced supply-chain limitations have now made ‘all-in-one’ commercial testing for COVID-19 the molecular norm in developed countries (leading to a gradual phase-out of CDC and similar hands-on assays), their associated costs can still be prohibitive in many settings. Several multiplex qRT-PCR assays have been developed by both commercial entities and diagnostic laboratories^16–19^, but none are based on the use of raw sample testing and many of these require specific reagents, instruments, and platforms outside of those required by widely used protocols promulgated by public health authorities^2^. As such, these assays are often more expensive to implement and are not as easily adapted into current laboratory workflows.

**Figure 7:**
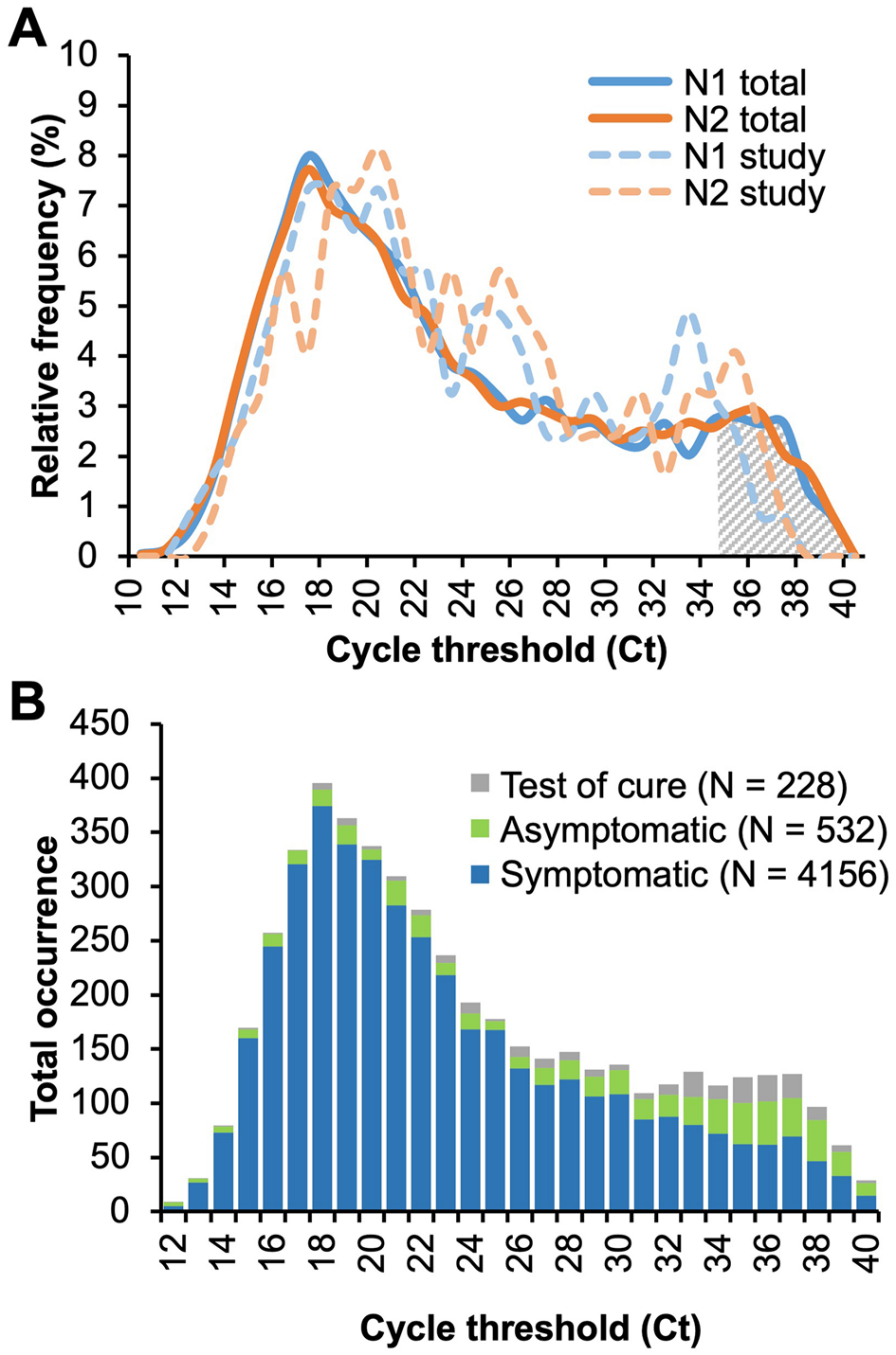
Comparisons of the C_t_ distributions for positive specimens performed in VUMC from Mar 2020–Mar 2021 using the CDC qRT-PCR. A) Ct distribution for N1 (solid blue line) and N2 (solid orange line) of total positives versus the N1 (dotted blue line) and N2 (dotted orange line) for the 41 samples used in this study. B) Distribution of the Ct values for all positive tests from three patient groups: symptomatic (blue), asymptomatic (green), and test of cure (grey).

We also observed that the majority of clinical samples we tested had C_t_s < 30 in the original CDC result. To ensure that the samples we tested were representative of the testing population, we compared the distribution of C_t_s among all the positive results detected by the Molecular Infectious Diseases Laboratory at VUMC over the five-month period our clinical testing was conducted to the distribution of the 41 positive samples selected for comparison studies. The mean N1 and N2 C_t_s for the CDC clinical testing population were 23.7 and 23.8 cycles, respectively (N = 4916). Figure 7A compares the distribution of N1 and N2 for the CDC tested clinical samples and subset of 41 positive samples tested without extraction. This shows that our clinical testing population was representative of the overall population being tested at VUMC.

Based on sensitivity of the assay relative to the original pool of ~ 5000 positive clinical C_t_ values, the cutoff of our assay for reliable SARS-CoV-2 detection is a C_t_ of about 35 cycles in the original gold standard CDC assay result (see grey crosshatched area in Fig. 7A). In the population of total positives at VUMC using the CDC assay, approximately 90% had C_t_ < 35 cycles. This indicates that if testing had been performed using our unextracted protocol, the overwhelming majority of samples would be under our assay cutoff for reliable detection. Thus, the sensitivity of 92.7% we found in our assay would likely be the same over the entire testing population, particularly for N1.

A retrospective examination of the patient breakdown of the total positive samples provides further insight into the appropriateness of certain use case scenarios for the optimized test. Given that the distribution of C_t_ values for positive tests from symptomatic patients is skewed toward lower C_t_ values, approximately 95% of them fell below the < 35 C_t_ cutoff (Fig. 7B, blue bars). On the other hand, approximately 72% of positive tests from asymptomatic patients and 71% from follow-up testing for patients that previously tested positive (i.e., “test of cure”) fell below the < 35 C_t_ cutoff (Fig. 7B, green and grey bars, respectively). These data suggest that the optimized assay would be most appropriate for symptomatic testing, where the sensitivity is predicted to be 95% or greater.

The purpose of the RNA extraction method is to maximize the delivery of the RNA in the clinical sample to the qRT-PCR reaction and, at the same time, to minimize adding “interferents” that may negatively affect the reaction performance. Therefore, the main drawbacks of this protocol are that without RNA extraction, the RNA sample is less concentrated (i.e., fewer copies of RNA are added to the reaction) and potential PCR interferents from the specimen are not removed. One of the reasons for performing the VTM volume experiments was to indirectly investigate these factors. Since we observed a consistent C_t_ increase of about 5 to 6 cycles for the three CDC primers sets, we sought to determine which of these factors contributed to this C_t_ increase. It is likely that the C_t_ delays observed with raw unextracted samples are partly a result of adding fewer RNA targets compared to the extraction workflow. Based on dilutions performed during the extraction step and comparing the minimum dilutions to the dilutions for unextracted samples, extracted samples have nearly three times the concentration of viral RNA as compared to that of unextracted samples. This estimate is based on the differences in the fractions of the initial RNA transferred to the qRT-PCR for the unextracted case and the extracted case. For the unextracted sample, the fraction is simply the fraction of the total sample volume added in a reaction. For a 3 mL VTM specimen where only 5 µL is added to the reaction, just 0.17% of the total available volume is tested in the unextracted method. The CDC’s extraction-based methodology, on the other hand, results in a greater amount of the VTM specimen in the reaction. Based on volumes used, the dilution factors, and an RNA extraction efficiency assumption, the extracted sample is a product of the fraction used in the extraction procedure (200 µL out of 3 mL is used) times the fraction of the final elution volume of the extraction volume added to the qRT-PCR reaction (5 µL out of 60 µL is used) times the extraction efficiency (reasonable to assume 70% efficiency). Based on volumes alone, the sample input is 0.56% of the total VTM volume, and including a 70% extraction efficiency, the sample input becomes 0.39% of the total VTM volume. This represents more than a two-fold increase in sample input for the extracted sample compared to the unextracted sample (0.17% and 0.39%, respectively). Depending on the reaction efficiency, this greater sample input can account for only about a 2 to 3 cycle difference in C_t_. The observed C_t_ difference of the unextracted assay is roughly five to six cycles and having three times more target present is insufficient to explain the ΔC_t_ change.

Since the difference in the number of targets is insufficient to explain the increase in C_t_, this suggests that direct transfer of a nasopharyngeal sample also adds one or more interferents to the qRT-PCR reaction. At an annealing temperature of 55 °C (i.e., the prescribed CDC annealing temperature), we found a C_t_ shift for the N2 primer set but little change for N1 when comparing the addition of water to various VTM cocktails (Fig. 1) and to changes in sodium (Fig. 3). However, at 61 °C this effect was not observed. In pooled patient specimens, N2 shifts compared to water were about 11.9 and 0.2 C_t_ at 55 °C and 61 °C, respectively (Fig. 2). This suggests that both a decrease in the number of targets and the addition of an unknown interferent from VTM contribute to the increase in C_t_ for all primers. At this point we have been unable to completely account for the increase in C_t_ that is seen across all primer sets. C_t_ differences might be improved by either incorporating a more robust polymerase that has a better performance in the presence of interferents combined with an increase in the VTM volume added to the PCR reaction mixture to increase the number of targets in the qRT-PCR reaction.

Although we were able to overcome the differences in N1 and N2 primer performance by modifying the anneal temperature, it remains unclear why the N2 primer set is negatively impacted when using direct transfer from VTM when the CDC anneal temperature is 55 °C (Figs. 1B, 2) or when larger volumes of VTM are used even at an annealing temperature of 61 °C (Fig. 4B). Since this effect is primer-specific, it is unlikely that it can be accounted for by temperature effects on the polymerase during the extension phase, since this would have altered the performance of both primer sets. Based on the IDT web tools, all the primers in the assay have similar melt temperatures, ranging from 53.6, 56.6, 53.7, and 55.9 °C for the forward N1 primer, reverse N1, forward N2, and reverse N2, respectively. Primer design remains an inexact process, and melt and anneal temperatures can be predicted based solely on GC content and the salt composition of the reaction^20^. However, due to unpredictable variables, these in silico primer design tools only serve as a guideline, and primer selection and qPCR cycling conditions are most often evaluated empirically. Our observations suggest that at least one of the N2 primers is not annealing at the melt temperature predicted based on GC content and [Na +]. We hypothesize that an unknown interferent is present in the final qRT-PCR reaction the either directly blocks annealing through a sequence-specific interaction with the N2 target or primers/probe or that indirectly prevents access to the N2 target region by inducing or stabilizing the formation of secondary or tertiary structures near the N2 target sequence. The mechanism by which increasing the annealing temperature from 55 to 61 °C enables efficient N2 amplification in the presence of a VTM specimen remains under investigation.

A small delay for RNase P was also observed, and this is most likely due to greater competition for reagents in multiplex reactions. During multiplex reactions, later amplifying reactions have less reagents available for use than those amplifying earlier. This is supported by the observation that this delay in C_t_ for RNase P did not occur in negative samples (Δ*C*_t_ = 0.63 ± 0.2), where only the RNase P target amplifies and there is no competition for reagents. Thus, this delay only affects positive samples where multiple targets are amplifying. Because the criteria for a positive result does not require RNase P amplification, categorical agreement between our protocol and the clinical result are not affected.

## Conclusions

Increasing the CDC SARS-CoV-2 annealing temperature to 61 °C and combining the original three singleplex reactions for N1, N2 and RNase P into a multiplex reaction produced a resource-optimized design for direct testing for SARS-CoV-2 from unextracted nasopharyngeal samples while retaining a categorical sensitivity of 92.7% and specificity of 100%.

In developing this CDC optimization assay, we discovered that direct testing for nucleic acids from a biological sample matrix should acknowledge the possibility that direct sample matrix added to a qRT-PCR reaction may alter the reaction performance, particularly the optimal annealing temperature for the primers. By including a matrix optimization step for the CDC SARS-CoV-2 primer sets, we have found that modifying the temperature protocol produced a consistent shift in C_t_ for all primer sets of about 5.4 cycles compared to the standard CDC protocol. This change is only partially explained by the theoretically predicted changes based on the threefold fewer targets delivered to the qRT-PCR reaction by directly adding a VTM sample to the reaction compared to performing the standard CDC workflow of RNA extraction and adding the extracted nucleic acids to the reaction.

The modified workflow described here optimizes the direct use of heat-inactivated nasopharyngeal samples with the widely used primer and probe sequences, with only minor modifications to current laboratory workflows. For this specific assay and in general, multiplexing and eliminating RNA extraction significantly reduce cost and turnaround time, conserve reagents, and increase testing throughput capacity. Although eliminating RNA extraction does affect the qRT-PCR reaction analytic performance, based on a typical C_t_ distribution from large-scale clinical testing, the diagnostic impact is expected to be minimal, particularly for symptomatic patients. This modified protocol may prove valuable for laboratories performing symptomatic COVID-19 testing that have the capability to perform qRT-PCR but that lack adequate access to extraction reagents and automated extraction instrumentation. In the future, the approach employed here provides a framework for those developing direct testing methods for other pathogen-analytes and biological matrices, beyond just COVID and this particular assay.

## Methods

### Samples from human subjects

All experiments involving residual clinical specimens were performed under the approval of, and in accordance with, the Institutional Review Board (IRB) at Vanderbilt University School of Medicine (protocol #201804). This protocol was granted with a waiver-of-consent as these are residual diagnostic specimens that were collected in the context of routine care and fully de-identified before use in this study.

### qRT-PCR reagents and reaction protocols

Primer–probe sets for the CDC N1, N2, and RNase P targets were obtained from Integrated DNA Technologies (IDT, Cat # 10006770), the same commercial source as clinical-use testing. For standard singleplex testing, individual reaction mixtures were formulated per Emergency Use Authorization (EUA) instructions using TaqPath 1-Step RT-qPCR master mix (Life Technologies, Cat # A15299). For each reaction, EUA parameters were applied for reverse transcription (25 °C—2 min, 50 °C—15 min, 95 °C—2 min) and thermocycling (45 cycles of 95 °C—3 s and 55 °C—30 s) on a Thermo Fisher Scientific QuantStudio 5 instrument. In addition to CDC primer sets, we also explored the behavior of several other published SARS-CoV-2 primers, including E and RdRp^3, 9, 21^, all obtained from IDT. For surrogate optimization studies, the SARS-CoV-2 synthetic RNA genome (Biodefense and Emerging Infections (BEI) Research Resources Repository, Cat # NR-52358) was employed as the amplification target and spiked into VTM or water as described below. To more closely approximate real-world conditions, additional studies employed positive COVID-19 nasopharyngeal specimens (derivatives of clinical care) that were spiked into pooled COVID-19-negative specimens in VTM. To enable multiplexing, probes for N1, N2, and RNase P were obtained from IDT in separate detection channels—FAM, HEX, and Cy5, respectively. The multiplex reaction was again performed using TaqPath 1-Step qRT-PCR Enzyme Mixture, and primers were combined into a single reaction with N1, N2 and RNase P primers at a final concentration of 500 nM and the probes at 125 nM. Cycle threshold (C_t_) was determined using the default parameters on the QuantStudio 5 software and recorded in each data file.

### VTM interferent study methods

In the standard clinical protocol, nasopharyngeal specimens for COVID-19 testing are obtained via flocked Dacron swabs, inoculated into 3 mL of VTM and tested within 24 h of storage at 4 °C. The latter medium (Remel M4RT) is comprised of Hanks balanced salt solution (HBSS), supplemented with proprietary concentrations of HEPES buffer (pH 7.3), phenol red, bovine serum albumin, gelatin, sucrose, L-glutamate, gentamicin, and amphotericin B. To assess interference from VTM components, the N1 and N2 reactions in standard master mix (i.e., target in water, simulating specimen extraction) were compared with four different formulations of spiked matrix: standard VTM, HBSS + phenol red, HBSS + NaHCO_3_, and HBSS + phenol red + NaHCO_3_. qRT-PCR was performed using the CDC’s prescribed cycling conditions.

### Optimization of primer annealing temperature in VTM

Synthetic SARS-CoV-2 RNA (BEI, Cat # NR-52358) was serially diluted (highest initial RNA concentration = 10^4^ copies/µl) into both water and VTM, and these solutions were added to remaining qRT-PCR master-mix components. To compare assay performance across annealing temperatures, singleplex gradient qRT-PCR was performed at two-degree intervals from 55 to 65 °C (BioRad CFX96). Temperature-dependent N1 and N2 C_t_-values were compared for the water *versus* VTM inocula. Similar experiments were performed with pooled “hot” SARS-CoV-2 positive specimens (~ 100 residual clinical VTM samples with very high virus content [C_t_s of 12 – 15 cycles]), which were diluted 100-fold into pooled negative matrix or water prior to direct inoculation into master mix and amplification (Thermo Fisher QuantStudio 5). Standard deviation was calculated and plotted as error bars.

### Evaluation of optimized conditions in the presence of sodium

To assess the specific effects of salt on the N1 and N2 reactions, VTM-relevant salt concentrations of 0 to 35 mM sodium chloride (Sigma Aldrich, Cat # S5150-1L) in reaction were spiked into an otherwise VTM/HBSS-free reaction and tested at 55 and 61 °C. The N1 and N2 reactions were performed independently using the TaqPath 1-Step qRT-PCR Master Mix (Life Technologies, Cat # A15299) and the 2019-nCoV CDC EUA Kit (IDT, Cat # 10006770) primer/probe N1 and N2 mixtures. Quantitative Synthetic RNA from SARS-Related Coronavirus 2 (BEI, Cat # NR-52358) at a concentration of 10^5^ copies/µL was diluted tenfold into each sodium solution to result in 50,000 copies per reaction.

### VTM volume optimization studies

To determine the optimal volume of VTM added to the reaction, a constant number (10^3^ copies/µl) of synthetic RNA targets from SARS-Related Coronavirus 2 (BEI, Cat # NR-52358) was added to a reaction mix containing either 0, 10, 25, or 42.5% VTM (0, 2, 5, or 8.5 µL, respectively). This latter value is the maximum allowable volume when replacing the water added to the reaction using the TaqPath kit. These reactions were performed at the 61 °C annealing temperature that was determined to be optimal.

### Comparison of singleplex and multiplex reactions

The primer–probe sets in singleplex and multiplex were tested in parallel and the C_t_ were compared. In these comparison tests, VTM was spiked with synthetic RNA (BEI, Cat # NR-52358). Synthetic RNA at a concentration of 10^2^ copies/µl spiked into VTM was added to each reaction. This design was also repeated using a pooled positive clinical specimen diluted into a pooled negative specimen instead of RNA spiked into VTM.

### Clinical sample selection, clinical lab CDC testing methods and comparison with optimized multiplex reaction

Fresh (never frozen) clinical nasopharyngeal specimens in VTM were obtained from the Molecular Infectious Diseases Laboratory at Vanderbilt University Medical Center (VUMC). Based on the CDC assay results in this CLIA-accredited laboratory, a total of 41 SARS-CoV-2 positive samples and 43 SARS-CoV-2 negatives were obtained. During sample collection, the patient’s nasopharyngeal swab was diluted into 3 mL containers of VTM. A total of 200 µL of VTM were processed to extract RNA, and the resultant viral RNA eluted into 60 µL of kit eluant buffer, of which 5 µL was added to each reaction. To perform comparison studies, samples from the clinical workflow were collected across a five-month period from November 2020 to March 2021 and the residual unextracted clinical samples were stored at 4 °C (< 24 h) until use. Residual nasopharyngeal samples were de-identified and 50 µL of each sample was placed on a pre-heated bench-top heat block set to 95 °C for 10 min in preparation for the unextracted assay. Five microliters of each were subsequently added to qRT-PCR reactions. Thermal cycling was performed using the following settings: 25 °C for 2 min, 50 °C for 15 min, 95 °C for 2 min, (95 °C for 3 s and 61 °C for 30 s) × 45 cycles. The C_t_s of the heat-treated unextracted and the original clinical assay result were obtained. Overall assay sensitivity and specificity was determined by categorical agreement between the original clinical result and the unextracted test result. For determining a positive result for inclusion in this study, categorical qRT-PCR results were interpreted using the guidelines set forth by the CDC protocol: A result is deemed positive if both N1 and N2 are detected with C_t_ < 40, and a result is deemed negative if neither N1 or N2 are detected and RNase P is detected with C_t_ < 40. Results are ruled indeterminate if i) only one of N1 and N2 are detected with C_t_ < 40, or ii) N1, N2, and RNase P are not detected. For the 41 positive and 43 negative specimens used in this study, categorical agreement and 95% confidence intervals were determined for each primer set individually.

## Supplementary Information

Supplementary Information.

## Data Availability

The datasets generated and analyzed for the current study are available from the corresponding author on reasonable request.
