# Supplementary material for: Direct PCR with the CDC 2019 SARS-CoV-2 assay: optimization for limited-resource settings
Source: Sci Rep. 2022 Jul 11;12:11756. doi: 10.1038/s41598-022-15356-7 (PMC9272867; doi:10.1038/s41598-022-15356-7)
Supplement: Supplementary file 1 — Supplementary Information. [file 41598_2022_15356_MOESM1_ESM.pdf]

Supplementary Materials

Direct PCR with the CDC 2019 SARS-CoV-2 Assay: Optimization for Limited-Resource Settings

Christia M. Victoriano[1], Megan E. Pask[1], Nicole A. Malofsky[1], Adam Seegmiller[2], Steve Simmons[3], Jonathan E. Schmitz[2,4,5], Frederick R. Haselton[1,6,#], Nicholas M. Adams[1,#]

Supplementary Figure 1

Supplementary Figure 2

Supplementary Figure 3

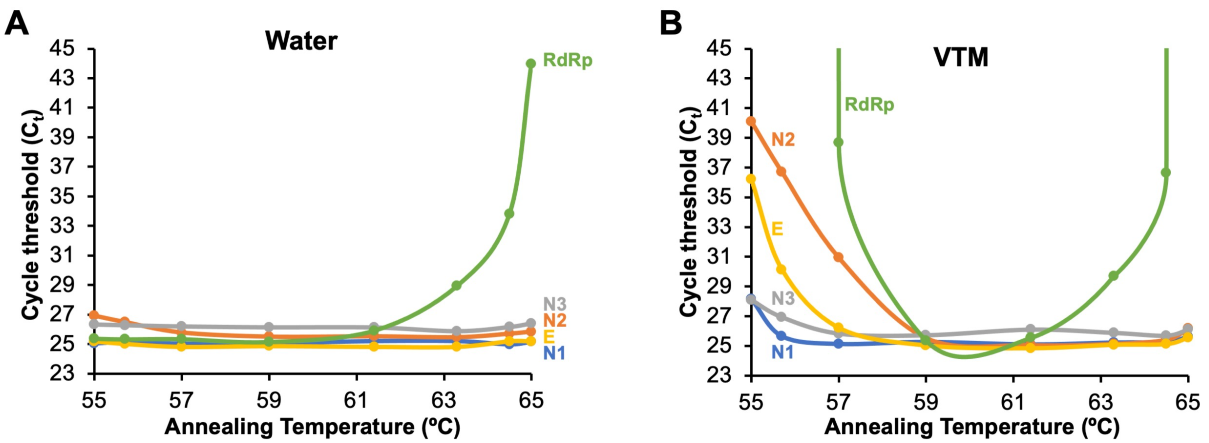

Supplement Figure 1. Effect of VTM addition on optimal primer annealing temperature of five common primer sets for SARS-CoV-2 detection. (A) With water added, C_ts for all targets, except RdRp, showed little to no variation across the range of annealing temperatures. The differences at 55 °C between water and VTM were 3.2 and13.2, for N1 and N2, respectively. At 61 °C these were reduced to -0.1 and -0.5. (B) With VTM added, C_ts for all five targets showed variation across the 10 °C range of annealing temperatures, particularly at 55 – 57 °C. The optimal annealing temperature in VTM was found to be 61 °C for N1 and N2, the primers used for the rest of these studies (N = 2).

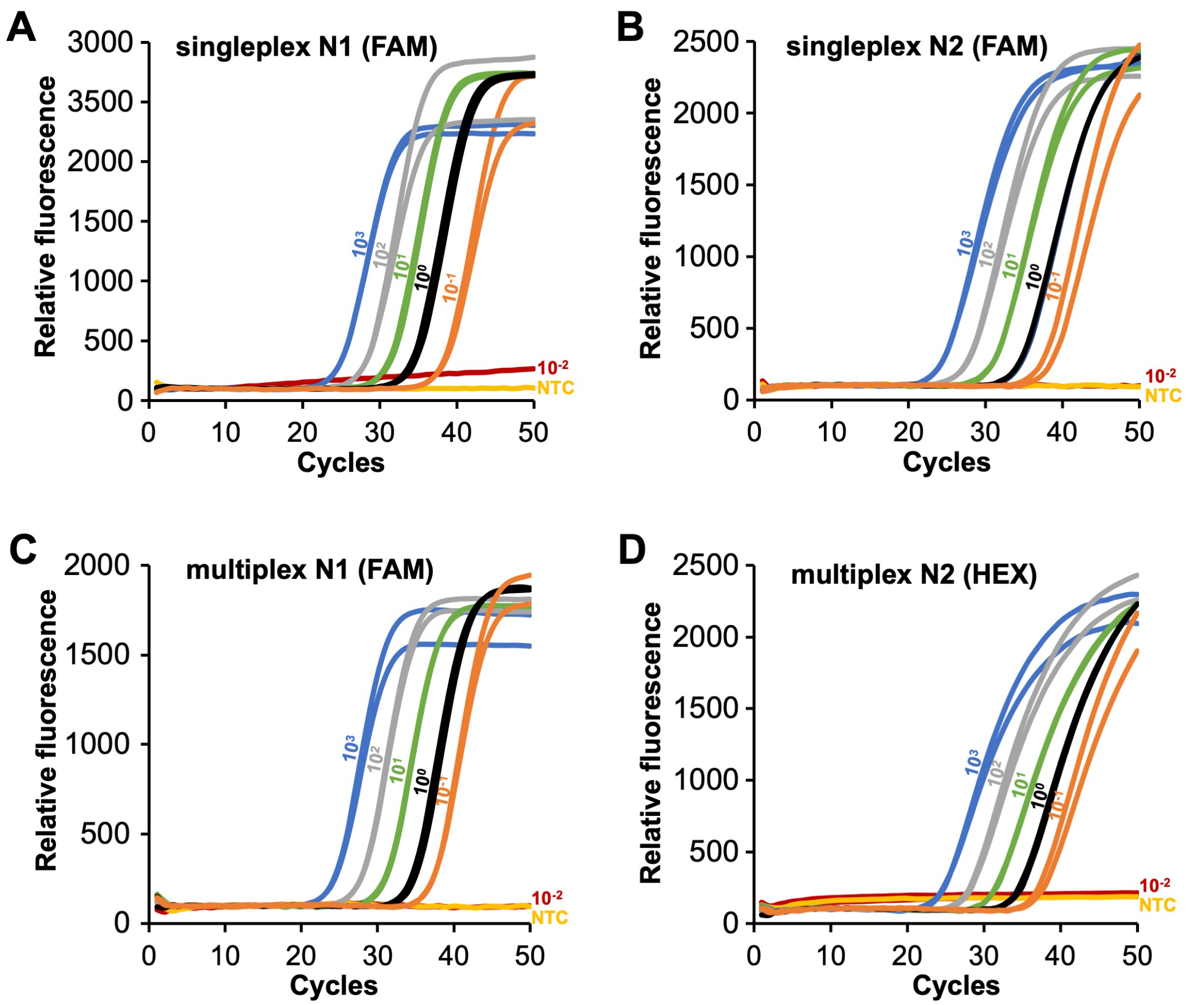

Supplemental Figure 2. Comparison of single-plex to multiplex qRT-PCR standard dilution series of synthetic RNA spiked in VTM at 61 °C annealing. Singleplex qRT-PCR curves for primer set N1 (panel A) and N2 (panel B) at target concentrations from 10^{-2} to 10^{3} copies/reaction are compared to multiplexed reaction using FAM (panel C) and HEX (panel D) probes (N = 2).

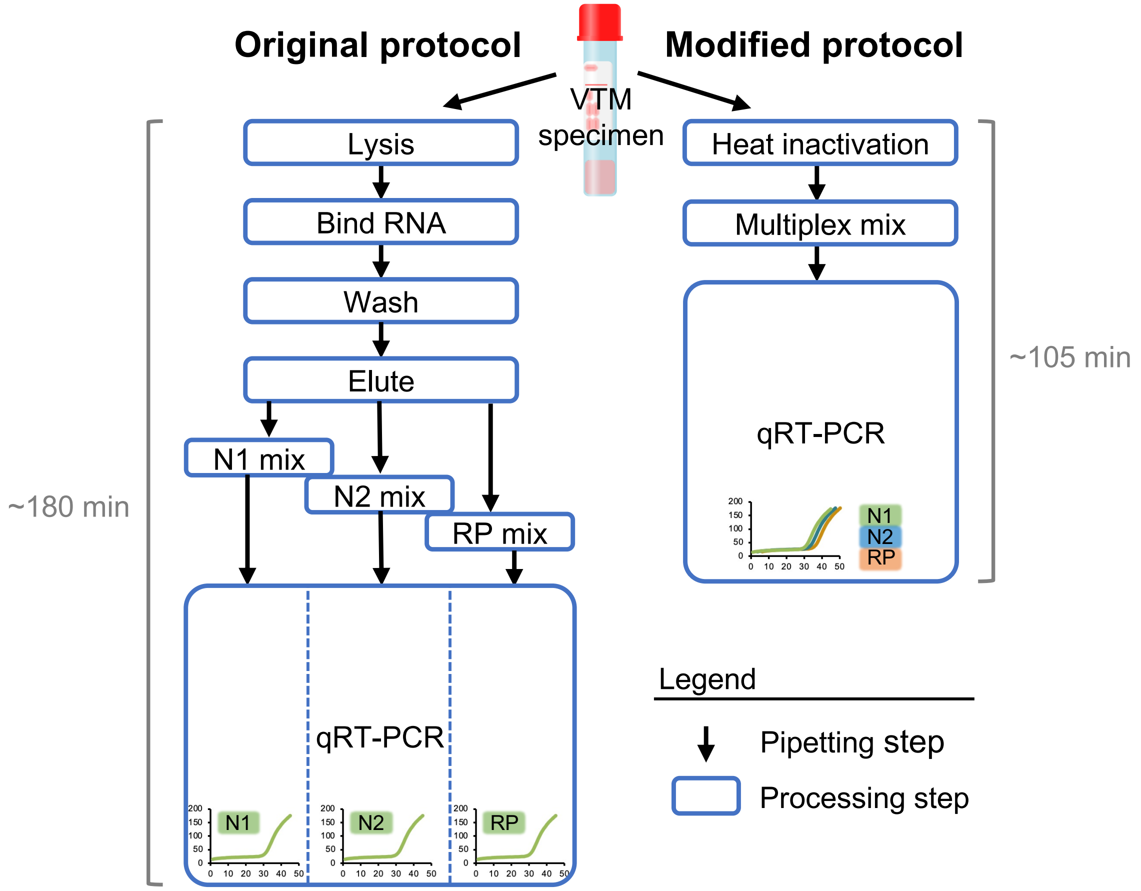

Supplemental Figure 3. Schematic comparison of the original CDC protocol and the modified direct qRT-PCR protocol.
